# Single-molecule real-time transcript sequencing facilitates common wheat genome annotation and grain transcriptome research

**DOI:** 10.1186/s12864-015-2257-y

**Published:** 2015-12-09

**Authors:** Lingli Dong, Hongfang Liu, Juncheng Zhang, Shuangjuan Yang, Guanyi Kong, Jeffrey S. C. Chu, Nansheng Chen, Daowen Wang

**Affiliations:** The State Key Laboratory of Plant cell and Chromosome Engineering, Institute of Genetics and Developmental Biology, Chinese Academy of Sciences, Beijing, 100101 China; Frasergen, Wuhan East Lake High-tech Zone, Wuhan, 430075 China; University of Chinese Academy of Sciences, Beijing, 100049 China; School of Pharmaceutical Sciences, Wuhan University, Wuhan, 430071 China; School of Life Science and Technology, Huazhong Agricultural University, Wuhan, 430075 China; Department of Molecular Biology and Biochemistry, Simon Fraser University, Burnaby, British Columbia Canada; The Collaborative Innovation Center for Grain Crops, Henan Agricultural University, Zhengzhou, 450002 China

**Keywords:** SMRT sequencing, PacBio RSII, Genome annotation, Transcriptome, Grain development, Common wheat

## Abstract

**Background:**

The large and complex hexaploid genome has greatly hindered genomics studies of common wheat (*Triticum aestivum*, AABBDD). Here, we investigated transcripts in common wheat developing caryopses using the emerging single-molecule real-time (SMRT) sequencing technology PacBio RSII, and assessed the resultant data for improving common wheat genome annotation and grain transcriptome research.

**Results:**

We obtained 197,709 full-length non-chimeric (FLNC) reads, 74.6 % of which were estimated to carry complete open reading frame. A total of 91,881 high-quality FLNC reads were identified and mapped to 16,188 chromosomal loci, corresponding to 13,162 known genes and 3026 new genes not annotated previously. Although some FLNC reads could not be unambiguously mapped to the current draft genome sequence, many of them are likely useful for studying highly similar homoeologous or paralogous loci or for improving chromosomal contig assembly in further research. The 91,881 high-quality FLNC reads represented 22,768 unique transcripts, 9591 of which were newly discovered. We found 180 transcripts each spanning two or three previously annotated adjacent loci, suggesting that they should be merged to form correct gene models. Finally, our data facilitated the identification of 6030 genes differentially regulated during caryopsis development, and full-length transcripts for 72 transcribed gluten gene members that are important for the end-use quality control of common wheat.

**Conclusions:**

Our work demonstrated the value of PacBio transcript sequencing for improving common wheat genome annotation through uncovering the loci and full-length transcripts not discovered previously. The resource obtained may aid further structural genomics and grain transcriptome studies of common wheat.

**Electronic supplementary material:**

The online version of this article (doi:10.1186/s12864-015-2257-y) contains supplementary material, which is available to authorized users.

## Background

Structural and functional genomics studies are fundamental to the understanding of plant biology. To effectively perform such studies, it is essential to have access to high-quality genome and transcriptome sequences. The advent of second generation sequencing (SGS) technologies, such as the Illumina technology, has stimulated the construction of genome and transcriptome resources for many plant species [1–4]. The development of a draft genome sequence using SGS technologies generally involves three main steps: generating and assembling short sequence reads into longer DNA contigs, ordering contigs along chromosomes, and annotating protein-coding genes and other elements for the contigs [1, 2]. The construction of transcriptomic sequences generally entails the production and assembly of short RNA-seq reads, and the assembly step can be made easier if there is a high-quality genome sequence available as a reference [3, 4].

During the development of genome and transcriptome resources, full-length transcripts can greatly increase the accuracy of genome annotation and transcriptome characterization when compared to the transcript tags assembled from short RNA-seq reads. Full-length transcript sequences permit efficient analysis of exon-intron structure and alternative splicing, thus facilitating a complete understanding of the transcriptional behavior of genomic loci [5–7]. Furthermore, the well characterized full-length transcripts are also beneficial for subsequent functional studies of important loci. On the other hand, the transcript tags derived from RNA-seq may suffer from misassembly of the reads transcribed from highly repetitive regions or very similar members of multigene families [3, 4]. This problem may become even more severe for polyploid plants that often harbor a large number of nearly identical homoeologous gene sets. Although full-length transcripts are highly desirable, their production is often labor intensive and time consuming in the past because of the need to clone individual cDNAs and to sequence them by traditional Sanger sequencing [5–7]. Recently, the third generation sequencing technology PacBio RSII has emerged as a unique opportunity for constructing full-length transcripts [8, 9]. This technology accomplishes single-molecule real-time (SMRT) sequencing with a read length up to 20 kb [8, 9], which renders PacBio RSII very effective in sequencing full-length cDNAs including long transcript isoforms [10–12]. One concern on PacBio sequencing is its relatively high error rate, but this can be effectively improved through implementing two types of corrections, i.e., by constructing consensus sequence reads from raw PacBio subreads and by alignment with the reads generated from appropriate SGS platforms [13–15]. However, compared to many successful applications in human research (http://www.pacificbiosciences.com/news_and_events/publications/), the use of PacBio sequencing to assist plant transcriptome investigations has so far been limited [16–20]. Nevertheless, a significant progress has recently been made on using SMRT cDNA reads to aid the prediction and validation of plant gene models [20].

Owing to broad adaptability and numerous end-uses, wheat is the most widely cultivated and consumed staple food crop [21]. Among the two main types of wheat cultivated currently, the hexaploid common wheat (bread wheat, *Triticum aestivum,* AABBDD, 2n = 6x = 42) is predominant, accounting for 95 % of global wheat production [22]. As a polyploid, the genome of common wheat is both large (about 17 G) and complex (containing 80 % or more repetitive DNA) [23, 24]. A complete reference genome sequence is still unavailable for common wheat. But a draft genome sequence, constructed mainly using Illumina HiSeq sequencing technology and covering about 60 % of the hexaploid genome, has recently been published for the spring-type common wheat land race Chinese Spring (CS) [24]. A large number of contigs were assembled for each of the 21 chromosomes, and a total of 133,090 high-confidence genes were annotated [24]. Because of its incomplete coverage, it is possible that many genes are missing or fragmented (i.e., exist in multiple short contigs) in the current draft genomic sequence. Thus, substantial efforts are being devoted to improve this draft genome sequence through refining contig assembly, gene annotation or both for individual chromosomes [25–27].

The A and D subgenomes of common wheat are derived from *Triticum urartu* (A^u^A^u^) and *Aegilops tauschii* (D^t^D^t^), respectively, whereas subgenome B is probably originated from an extinct *Aegilops* species [28, 29]. A natural hybridization, occurred about 10,000 years ago between wild tetraploid wheat (*T. turgidum* ssp. *dicoccoides*, AABB) and *Ae. tauschii*, gave rise to common wheat [29]. Thus, the A and D subgenomes of common wheat are largely syntenic with their counterparts in the diploid progenitors [29]. Using Illumina HiSeq platform, draft genome sequences have been developed for *T. urartu* and *Ae. tauschii*, with the number of protein-coding genes annotated for the two species being 34,879 and 43,150, respectively [30, 31]. There is also an effort to develop a detailed genomic sequence of *Ae. tauschii* based on sequencing chromosomally ordered BAC clones [32], http://aegilops.wheat.ucdavis.edu/ATGSP/data.php]. The contigs and annotated gene sets of *T. urartu* and *Ae. tauschii* have been useful for constructing the draft genome of common wheat [24]. In addition, the genomic information of other sequenced grasses, including rice, *Brachypodium distachyon*, sorghum and maize, has also aided contig ordering and gene annotation during developing CS draft genome sequence [24].

Grains represent the most valuable organ of common wheat, and have been the subject for numerous genetic, breeding, and more recently, functional genomics studies [32, 33]. They develop after double fertilization of caryopses, and their morphometric, weight and biochemical traits are the targets of wheat yield and quality improvement efforts [32, 33]. A huge number of genes and genetic interactions have been found involved in common wheat grain development and their function is often regulated at multiple levels, including temporal and spatial transcriptional regulations [32, 33]. Because of this complexity, our understanding of the genes functioning in common wheat grain development is still incomplete, which is not conducive for effectively improving the yield and end-use traits of this important crop.

The main objectives of this study were to sequence the transcripts expressed during common wheat caryopsis development using the emerging SMRT sequencing platform PacBio RSII, and to assess the resultant data for improving common wheat genome annotation and grain transcriptome research. Towards these aims, we first identified a population of full-length non-chimeric (FLNC) SMRT cDNA reads from a pooled sample of unfertilized caryopses and developing grains using PacBio sequencing. Then we mapped the reads to the draft genome sequence of CS, and performed an in-depth analysis of the high-quality reads. Finally, we examined the value of the FLNC reads for finding full-length transcript sequence of the genes encoding three complex families of gluten proteins: high-molecular weight glutenin subunits (HMW-GSs), low-molecular weight glutenin subunits (LMW-GSs) and gliadins. These proteins are specifically expressed in the developing grains and are important determinants of the processing and nutritional qualities of common wheat [34]. We also produced transcriptomic reads using HiSeq 2000 for the unfertilized caryopsis and developing grain samples, separately, for three purposes, i.e., error correction of FLNC reads, validation of exon-intron junction sequence in FLNC reads, and investigation of the genes whose transcription was differentially regulated during caryopsis development. The experimental variety used in this work, Xiaoyan 81, is an elite winter-type common wheat line with super end-use quality [35]. Understanding the transcripts during the grain development of Xiaoyan 81 may provide useful transcriptome resource for genetically enhancing the end-use quality of common wheat.

## Results

### Transcript sequencing and error correction

Using mRNAs extracted from a pooled sample of unfertilized caryopses and developing grains collected at 5, 15 and 25 days after anthesis (DAA), two different libraries, with cDNA insert size < 2 kb and ≥ 2 kb, respectively, were prepared. Each library was sequenced using four SMRT cells on PacBio RSII platform. In total, we obtained 526,915 continuous long reads (CLRs), including 265,832 from the short-insert library and 261,083 from the long-insert library (Additional file 1). Following a previous publication [20], the CLRs were divided into two types, type I containing two or more subreads and type II with only one subread. From type I CLRs we identified a total of 1,618,400 subreads, which formed 240,312 circular consensus sequences (CCSs) after merging and error correction through subread comparison (Additional file 1). Of the 240,312 CCSs, 175,375 were found to be FLNC reads because each of them contained a distinct poly(A) tail and the 5′ and 3′ cDNA synthesis primers. The type II CLRs carried 570,293 subreads, from which 22,334 FLNC reads were identified (Additional file 1). Consequently, a total of 197,709 FLNC reads (175,375 + 22,334) were obtained.

To make further correction of the 197,709 reads, we generated Illumina HiSeq 2000 transcriptomic reads for each of the unfertilized caryopsis and developing grain samples. After adaptor sequence trimming and low-quality read filtering, we obtained averagely 69.3 million reads (with a mean size of 101 bp) for each of the four samples (Additional file 2). The proovread software, which had been found highly efficient for correcting SMRT sequences through iterative short read consensus [20, 36], was used to correct 197,709 reads. Before proovread correction, the average alignment identity of the 197,709 reads to CS draft genome sequence was 96.2 %. This value was increased to 98.3 % after proovread correction. We therefore focused our subsequent investigations on the 197,709 error corrected FLNC reads.

### Estimation of the proportion of FLNC reads carrying complete open reading frame

To evaluate the proportion of FLNC reads carrying complete open reading frame (ORF), we made use of the 5495 wheat full-length cDNAs published previously [37] and the draft genome sequence of CS. After mapping wheat full-length cDNAs and FLNC reads to the draft genome using the software GMAP [38], 1347 gene loci were found to be covered by both data sources. The number of FLNC reads assigned to the 1347 loci was 28,599. Out of the 28,599 reads, 21,326 (74.6 %) carried complete ORF (with start and stop codons) as defined in the wheat full-length cDNAs.

### Genome mapping of 197,709 FLNC reads

**Fig. 1:**
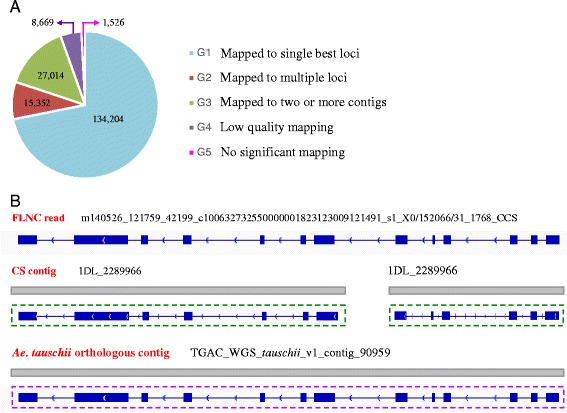
Mapping FLNC reads to the draft genome sequence of Chinese Spring (CS). **a** Division of the 186,765 FLNC reads into five groups (G1 to G5) based on their genome mapping characteristics. The number of reads in each group is depicted in the pie chart. **b** An example illustrating the FLNC read mapped to two different CS contigs located on the same chromosome arm. The read is shown as a split-mapped molecule (SMM) with both exon (filled box) and intron (line between two neighboring exons) depicted. The arrow indicates the direction of alignment to the genomic sequence. The two CS contigs to which the shown FLNC read mapped were both located on the long arm of chromosome 1D (1DL). The representative transcripts annotated for the two contigs by the draft genome sequence of CS are shown below as SMMs (boxed in green). The bottom panel is the *Ae. tauschii* contig orthologous to the two 1DL contigs of CS. The transcript annotated for this *Ae. tauschii* contig is also provided as a SMM (boxed in purple). The *Ae. tauschii* contig was identified by mapping the exemplary FLNC read to the D^t^ genome sequencing database (http://aegilops.wheat.ucdavis.edu/ATGSP/data.php). The diagrams shown are not drawn to scale

First, the 197,709 FLNC reads were mapped against the draft genome sequence of CS using GMAP. During mapping, the alignment direction of 10,944 reads could not be reliably determined. Therefore, the genome mapping characteristics of the remaining 186,765 reads were further investigated. These reads could be divided into five groups (G1 to G5, Fig. 1a). G1 consisted of 134,204 reads (67.88 % of the total), each of which could be mapped to one unique location with higher than 90 % coverage and identity. Thus, the G1 reads were mapped to the draft genome sequence with high-confidence. G2 contained 15,352 reads showing multiple best alignments (with identity and/or coverage values ≥ 90 %). G3 included 27,014 reads exhibiting partial mapping to two or more distinct draft genome contigs (coverage 30–80 %, identity ≥ 90 %). G4 had 8669 reads generally showing low-quality alignment to the draft genome (coverage 30–50 %, identity 40–90 %). Finally, G5 contained 1526 reads with no significant mapping to the draft genome.

Second, we examined the level of splice junction support with HiSeq transcriptomic reads for the well mapped G1 FLNC reads. As anticipated, nearly all of the splice junction sties (96 %) in the 134,204 G1 reads were supported by HiSeq transcriptome data. The most frequent splice junction was GT-AG (accounting for 98.51 %), followed by GC-AG (1.29 %), AT-AC (0.11 %) and some other minor types (0.09 %). These findings conformed to the consensus splice sites of higher plants [39–41].

Third, we investigated mapping characteristics of the reads in G2 and G3. Among the 15,352 G2 reads, a few (303) exhibited less than 90 % identity or coverage values when mapped to CS draft genome sequence, and were not considered further. Of the remaining 15,049 G2 reads, 554 showed one best mapping in all three (A/B/D) or two (A/B, B/D or A/D) subgenomes; 6348 exhibited mapping to two or more chromosomal locations in one of three subgenomes; 8147 were mapped to two or all three subgenomes with multiple mapping (≥2) found in at least one subgenome (Additional file 3). Therefore, the G2 reads were most likely transcribed from highly similar homoeologous and/or paralogous loci. Among the 27,014 G3 reads, 6102 were mapped to two different contigs, whereas the remaining 20,912 reads displayed alternative and complex mapping patterns. Interestingly, of the 6102 reads, 4219 bridged different CS contigs generally from the same chromosome arm (Fig. 1b), suggesting that the genes giving rise to the 4219 reads may not be adequately covered by the current CS draft genome sequence. To investigate this possibility, these reads were compared to the D^t^ genome sequence of *Ae. tauschii* constructed based on a high-quality physical map and BAC clone sequencing (http://aegilops.wheat.ucdavis.edu/ATGSP/data.php) [42]. We found 90 different cases where the two separate CS draft genome contigs bridged by a G3 FLNC read were actually contiguous in the D^t^ genome sequence (Fig. 1b, Additional file 4). A total of 200 similar cases were found when the 4219 G3 reads were compared to the scaffolds of *T. urartu* draft genome sequence (Additional file 4). Clearly, many of the G3 reads were likely useful for improving the contig assembly of CS draft genome sequence.

Lastly, because the FLNC reads in G4 and G5 showed low-quality or no apparent mapping to CS draft genome sequence, we investigated if some of them might have corresponding orthologous genes in the A^u^ genome of *T. urartu* or the D^t^ genome of *Ae. tauschii*. These reads were therefore aligned to the annotated protein-coding genes of *T. urartu* (34,879) and *Ae. tauschii* (43,150) [30, 31]. Assuming that some of the FLNC reads were mapped to distinct genes in the A^u^ or D^t^ genomes with both coverage and identity values higher than 90 %, then it might be possible that the orthologous common wheat loci do exist but are inadequately, or not yet, covered by the current draft genome sequence. As displayed in Additional file 5, the G4 FLNC reads having corresponding genes in *T. urartu* and *Ae. tauschii* amounted to 3076 and 2150, respectively, and a number of G5 reads were also found to have corresponding genes in both species. These data supported the assumption that many gene loci were still missing in the present draft genome sequence of CS.

### Detailed characterization of G1 FLNC reads

**Fig. 2:**
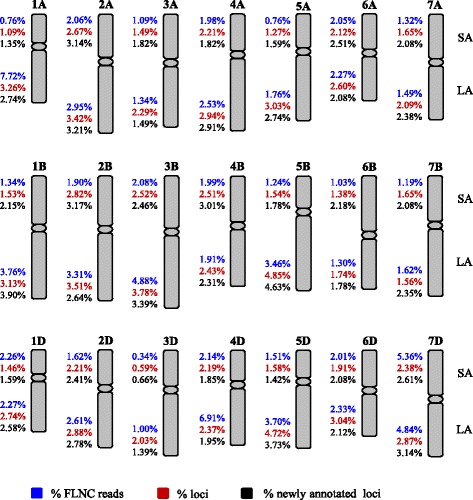
Chromosomal distributions of 91,881 high-quality FLNC reads and the loci identified by them. The known and new loci identified by the 91,881 reads were 16,188 and 3026, respectively. The three values were used as backgrounds for calculating the percentages displayed along each short arm (SA) and long arm (LA)

For further analyzing the 134,204 well mapped G1 FLNC reads, more stringent criteria were adopted during finding their genomic loci in CS draft genome sequence using GMAP. The reads missing 5′ exons and the singleton reads not supported by RNA-seq data were not considered further. These criteria were also applied in previous PacBio transcriptome studies [e.g., 10, 20]. With this filtering step, 42,323 FLNC reads were excluded from further analysis. The remaining 91,881 reads were regarded as having high-quality nucleotide sequence suitable for more detailed analysis. Of the 91,881 reads, 83,736 were assigned to 13,162 extant loci, and 8145 were mapped to 3745 contig regions that did not have prior annotated gene models (Table 1). The new loci defined by the 8145 reads amounted to 3026 (Table 1, Additional file 6). Of these newly identified loci, 666 were located on the contigs with other gene models, and 2360 were on the contigs that did not have any previously annotated gene models (Additional file 6). When compared to the genes in GO, KEGG, KOG and NR databases, 2433 of the 3026 new loci (80.4 %) could be annotated (Additional file 6). Together, the 91,881 reads were mapped to 16,188 loci distributed on 21 common wheat chromosomes (Table 1, Fig. 2). The percentages of mapped FLNC reads and loci varied among the 42 chromosomal arms (Fig. 2). The distribution of the 3026 new loci also differed among the 42 chromosomal arms, with more of them tending to be found on the long arms of subgenome B chromosomes (Fig. 2). Among the 16,188 loci, the majority (92.0 %) were covered by 2 to 10 FLNC reads, while the rest (8.0 %) were each supported by ≥ 10 reads. There were 49 loci each covered by higher than 100 reads.

**Table 1:** Genomic loci and unique transcripts represented by 91,881 high-quality FLNC reads

	FLNC read	Locus	Transcript
	83,736	13,162 (extant)	19,023 (13,177 extant, 5846 new)
	8145	3026 (new)	3745 (new )
Total	91,881	16,188	22,768

Unique transcripts represented by the cDNA inserts of 134,204 high-quality FLNC reads, including different isoforms for multi-exon genes, were examined. In this study, we defined the transcript isoforms of a multi-exon gene as having at least one different intron/exon junction. A total of 22,768 unique transcripts were identified, including 19,023 transcribed from previously annotated loci and 3745 from the loci newly annotated by this work (Table 1). Of the 19,023 transcripts corresponding to known loci, 13,177 confirmed previous annotations, whereas 5846 were newly discovered by this work (Table 1). Consequently, the total number of newly discovered transcripts by this work was 9591 (5846 + 3745, Table 1). The size of the 9591 transcripts varied from 575 to 4537 bp, with the mean being 2433 bp. For comparison, we calculated the size range and average length of previously reported transcripts for the 13,162 existing loci, which was 671–4636 bp with an average of 2388 bp. Thus, on average, the newly discovered transcripts were 45 bp longer than previously reported common wheat transcripts. The 22,768 transcripts identified based on our PacBio sequencing and their corresponding genomic loci and representative FLNC reads are listed in Additional file 7.

**Fig. 3:**
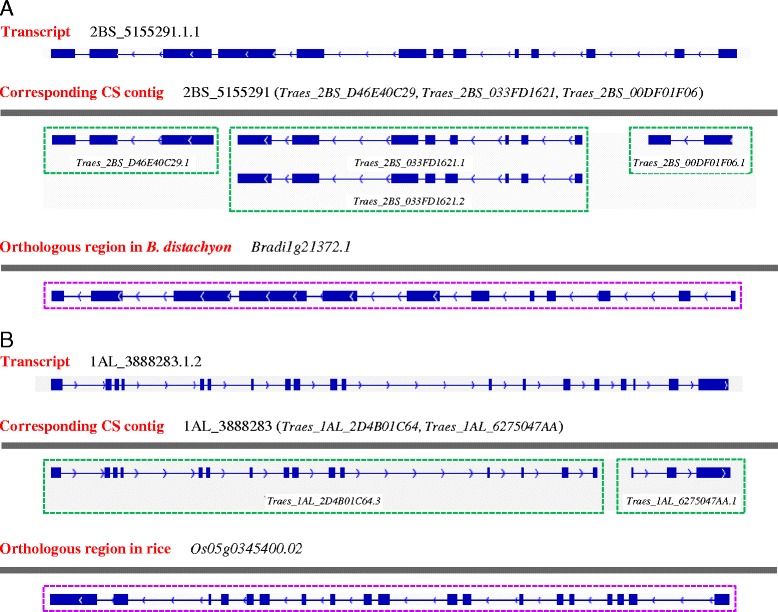
Analysis of representative transcripts spanning two or three Chinese Spring (CS) loci. **a** The transcript 2BS_5155291.1.1 and the three CS loci (*Traes_2BS_D46E40C29*, *Traes_2BS_033FD1621* and *Traes_2BS_00DF01F06*) it covered. These loci are located on the CS contig 2BS_5155291, and the transcripts annotated for the three loci by the draft genome sequence are boxed in green. The bottom panel shows *B. distachyon* genomic region orthologous to 2BS_5155291. A single locus (*Bradi1g21372.1*) and a corresponding transcript (boxed in purple) are annotated for this *B. distachyon* genomic region (http://www.plantgdb.org/BdGDB/). **b** The PacBio transcript 1AL_3888283.1.2 and the two CS loci (*Traes_1AL_2D4B01C64* and *Traes_1AL_6275047AA*) it covered. The two loci reside on the CS contig 1AL_3888283, and the representative transcripts annotated for them by the draft genome sequence are boxed in green. The bottom panel is the rice genomic region orthologous to 1AL_3888283. A single locus (*Os05g0345400.02*) and a corresponding transcript (boxed in purple) are annotated for this rice genomic region (http://www.plantgdb.org/OsGDB/). The transcripts in (**a** and **b**) are all shown as SMMs with exon (filled box) and intron (line between two neighboring exons) depicted. The diagrams are not scaled

Interestingly, we found 180 transcripts (among the 22,768 transcript set) each spanning two or three different genes annotated by CS draft genome sequence (Additional file 8). These transcripts carried intact ORF capable of encoding the polypeptides with 80 to 1307 amino acids (Additional file 8). One possible explanation for this observation was that the loci transcribing the 180 transcripts were incorrectly annotated into separate genes in CS draft genome sequence. To investigate this possibility, the 180 transcripts were mapped against the genome of *B. distachyon* and rice. The mapping results showed that 66 such transcripts were reliably mapped to discrete genomic locations each with a single gene annotation in *B. distachyon* (coverage ≥ 90 %, mean identity 87 %) (Fig. 3a). Twenty-eight of these transcripts could be aligned to distinct rice loci (coverage ≥ 90 %, mean identity 84 %), all of which had one annotated gene (Fig. 3b).

### Investigation of the genes differentially expressed during caryopsis development

To promote use of the transcripts identified by our PacBio sequencing for studying wheat caryopsis development in further research, we conducted the following lines of analysis. First, we computed the number of expressed genes, and the sum of expressed genes with coverage by PacBio sequencing identified transcripts, in the unfertilized caryopses and the developing grains at 5, 15 and 25 DAA (Table 2). The genes expressed at the four stages (S1 to S4), as identified using the uniquely mapped Illumina transcriptomic reads, amounted to 50,650, 42,444, 44,547 and 37,369, respectively (Table 2). These numbers were comparable to those reported for CS developing grains by a previous study [33]. At S1 to S4, the expressed genes with coverage by the transcripts identified through PacBio sequencing were 11,798, 9452, 10,626 and 8676, respectively, with the total number of such transcripts detected at the four stages being 17,330, 13,938, 15,909 or 12,943 (Table 2).

**Table 2:** Estimation of the genes expressed in unfertilized caryopses and developing grains

	Developmental stage^a^			
	S1	S2	S3	S4
Total number of genes expressed^b^	50,650	42,444	44,547	37,369
Number of genes with coverage by PacBio sequencing identified transcripts	11,798	9452	10,626	8676
Total number of PacBio sequencing identified transcripts detected at each stage	17,330	13,938	15,909	12,943

^a^S1, unfertilized caryopses; S2-S4, developing grains at 5, 15 or 25 days after anthesis

^b^Judged based on RPKM (reads per kilobase per million mapped reads) > 1

**Fig. 4:**
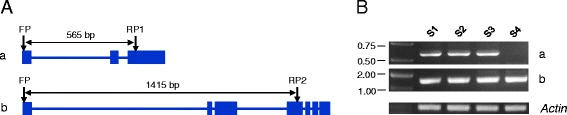
Analysis of the two different transcript isoforms of *TRAES_1DS_114C78BF4* by RT-PCR. **a** A diagram showing the exon (filled box)-intron (line in between filled boxes) patterns of the two isoforms (designated as a and b, respectively). Arrows mark the positions of the primers (FP, RP1 and RP2) used for specifically amplifying each of the two isoforms. The length of the amplicon (bp) is indicated for each isoform. **b** The result of amplifying isoforms a and b by RT-PCR in the caryopsis samples of four developmental stages (S1 - S4). Amplification of the common wheat *actin* gene (GenBank accession AB181991) served as internal control for normalizing cDNA content prior to PCR amplification. S1, unfertilized caryopses; S2-S4, developing grains collected at 5, 15 or 25 DAA. The data displayed are typical of three independent experiments

Second, we found 6030 genes showing differential expression of the transcripts identified by our PacBio sequencing among S1 to S4 (Additional file 9). Of these genes, 4783 had only a single form of transcript detected, and 1247 had two or more transcript isoforms. Two genes, *TRAES_1DS_114C78BF4* (encoding a putative RING/U-box superfamily protein) and *TRAES_2BS_ CDE410A7D* (specifying a probable O-fucosyltransferase family protein) were chosen as representatives to test if the differential expression computed may be verified by RT-PCR. Two different transcript isoforms (designated as a and b) were identified for *TRAES_1DS_114C78BF4*, with both present from S1 to S3 but only isoform b at S4 (Additional file 9). Consistent with this finding, RT-PCR analysis using specific primers confirmed the presence of both isoforms from S1 to S3 but only isoform b at S4 (Fig. 4). The two transcript isoforms (a and b) of *TRAES_2BS_CDE410A7D* were both detected at S1, with only one isoform found at S2 and S3 and no isoform expressed at S4 (Additional file 9). This differentially regulated isoform expression pattern was also confirmed by RT-PCR (data not shown).

### Finding of full-length transcripts for wheat gluten gene members

To test utility of the 197,709 FLNC reads, we used this resource to search full-length transcripts for the genes encoding three families of gluten proteins, i.e., HMW-GSs, LMW-GSs and gliadins (see Introduction). A common characteristic of the three gluten gene families is the presence of multiple homoeologous and paralogous copies with high sequence similarity [34], and hence the construction of full-length transcripts for these genes using short SGS reads is prone to misassembly. In our test variety Xiaoyan 81, there are three homoeologous loci (*Glu-A1*/*B1*/*D1*) carrying six closely related, single-exon *HMW-GS* genes, including five active members (*1Ax1*, *1Bx14*, *1By15*, *1Dx2* and *1Dy12*) and one pseudogene (*1Ay*) [35]. The *A*, *B* and *D* copies are homoeologs whereas the *x* and *y* members are paralogs. In our search, we detected 574 FLNC reads carrying complete *HMW-GS* ORF, and their corresponded well to six different full-length *HMW-GS* transcripts (Additional file 10). The *1Ay* alleles in common wheat and related species are highly similar, and have often been found to carry premature stop codon in the coding region [43, 44]. Consistent with the past observation, we noticed that the *1Ay* transcript sequence of Xiaoyan 81 was more than 99 % identical to two previously reported inactive *1Ay* alleles, and that the three alleles all carried a premature stop codon at the same position of the coding region (Additional file 11). The genes encoding LMW-GSs in common wheat come from three large homoeologous loci (*Glu-A3*/*B3*/*D3*) [34]. The number of *LMW-GS* genes in Xiaoyan 81 is still unknown, but its parental variety (Xiaoyan 54) has been found to carry 14 such genes (including 11 active and three pseudogene members) by a combined genomic and proteomic analysis [45]. Here, we found 139 FLNC reads carrying complete *LMW-GS* ORF, and they represented 14 distinct full-length *LMW-GS* transcripts (including 12 with intact ORF and two with disrupted coding region, Additional file 10).

The genes encoding common wheat gliadins, contained mainly in two sets of compound homoeologous loci (*Gli-A1*/*B1*/*D1* and *Gli-A2*/*B2*/*D2*), are exceedingly complex, and have been divided into three main subfamilies according to their protein products (α/β-, γ- or ω-gliadins) [34]. However, the exact numbers of genes coding for α/β-, γ- and ω-gliadins are still unclear in common wheat. Here, we detected 263 FLNC reads harboring complete α/β-gliadin gene ORF, and they identified 32 unique full-length transcripts for α/β-gliadins (including 25 with intact ORF and seven with disrupted coding region, Additional file 10). A total of 208 FLNC reads carrying complete γ-gliadin gene ORF were detected, and they represented 14 distinct full-length transcripts for γ-gliadins (12 with intact ORF and two with disrupted coding region, Additional file 10). This finding agreed with the previous annotation of 13 γ-gliadin coding genes for CS [46]. Lastly, 27 FLNC reads carrying complete ω-gliadin gene ORF were scored, and they corresponded to six different full-length transcripts for ω-gliadins, four of which had intact ORF (Additional file 10). The finding of six unique full-length transcripts for ω-gliadins here was consistent with the identification of five to seven ω-gliadin proteins in the grains of American and British common wheat cultivars [47, 48]. In total, we found 72 non-redundant full-length transcripts for HMW-GSs, LMW-GSs and gliadins (Additional file 10). The total positive FLNC reads found in this search were 1577, 1211 of which contained complete ORF (though some of them were disrupted). Thus, the proportion of FLNC reads with complete gluten gene ORF was thus 76.8 %.

## Discussion

In this work, we applied PacBio sequencing to investigate transcripts in the unfertilized caryopses and developing grains of common wheat. Following the latest methodologies in analyzing PacBio transcriptome data [10–12, 20, 36], we obtained 197,709 error corrected FLNC reads, 91,881 of which were found to be of high-quality. The new resource and transcriptional information gathered and their values for improving common wheat draft genome annotation and grain transcriptome research are discussed below.

### Utility of PacBio transcriptome sequencing for obtaining full-length transcript sequence information in plants

Full-length transcript sequence information is very useful for both genome annotation and gene function studies in plants. However, it is often difficult to obtain such information efficiently using traditional cDNA cloning and sequencing approaches (5–7). Here we suggest that PacBio sequencing is an effective route for obtaining reliable full-length transcript sequence information in plants, particularly for the polyploid species like common wheat. This suggestion is supported by the following evidence. First, about 74.6 % of the FLNC reads generated in this work were found to carry complete ORF when compared to several thousands of full-length cDNAs published previously. Second, by searching the 197,709 FLNC reads, we identified full-length transcripts for 72 transcribed gluten gene members belonging to three complex gene families, and the proportion of FLNC reads with complete gluten gene ORF was 76.8 %. Third, Our PacBio sequencing correctly captured the transcripts derived from a number of pseudogenes, such as *1Ay*, *Glu-D3-5* and the inactive gliadin gene members (Additional file 10). The sequencing of RT-PCR amplicons using PacBio platform by a previous study had also identified the transcripts from a number of gluten pseudogenes [18]. Together, these data provide good support for the reliability of the full-length nucleotide sequence information obtained through PacBio transcriptome sequencing. The self-correction through subread comparison and the additional correction using HiSeq transcriptomic reads have both contributed to yielding reliable nucleotide sequence data in PacBio sequencing. Finally, the construction of PacBio CCSs and FLNC reads completely avoided the need to assemble short transcriptomic reads. This advantage has enabled us to obtain and differentiate the full-length transcripts of three families of gluten genes with highly similar homoeologous and paralogues members (Additional file 10). Because the expression of homoeologous gene set with nearly identical gene members is a fundamental characteristic of polyploid plants, PacBio sequencing should be particularly valuable for transcriptome studies of these species.

The high capacity of PacBio transcriptome sequencing to generate full-length transcript sequence information may well be related to its long-read property. In agreement with this reasoning, we found that the 9591 newly discovered transcripts by our PacBio sequencing were on average more than 45 bp longer than the known transcripts of 13,177 existent loci. Previous transcriptome studies have also reported that PacBio sequencing represented an efficient strategy for identifying full-length and relatively long transcript sequences in human cells [10–12].

### Improvements on common wheat draft genome annotation

The large and complex hexaploid genome makes it difficult to develop a complete and high-quality reference genome sequence for common wheat in a short time. The available draft genome sequence of CS represents an important aid to common wheat structural and functional genomics investigations [49], and continuous refinement of the draft sequence with transcriptome data should enhance its utility. In this work, we annotated 3026 new chromosomal loci in the draft genome contigs based on newly gathered transcript evidence (Additional file 6). More than 80.4 % of the newly annotated genes had homologs in various databases, and thus represent a useful addition to the gene complement of the draft genome sequence. We found 9591 new transcripts (Additional file 7), which not only enrich the transcriptional information of the draft genome sequence but also are useful for functional studies of important genes in further research (see also below). Two other sets of data with direct implications for future revision of the draft genome sequence are 1) the finding of 290 G3 FLNC reads each mapped to two draft genome contigs from the same chromosome arm (Additional file 4, Figs. 1b and 2) the observation of 180 transcripts each spanning two or three previously annotated gene models (Additional file 8, Fig. 3). The 290 FLNC reads should be useful for refining chromosomal contig assembly, whereas the 180 transcripts can assist more detailed annotations of the concerned chromosomal loci.

In addition to the improvements discussed above, the 15,352 G2 FLNC reads with three patterns of multiple genome mapping (Additional file 3) are potentially useful for annotating highly similar homoeologous or paralogous genes of common wheat. The FLNC reads in G4 and G5 may aid future annotation of the chromosomal loci not covered by the current draft genome sequence. There is also a possibility that some of the FLNC reads in G4 and G5 may come from divergent variety-specific genes since the elite common wheat variety used here (i.e., Xiaoyan 81) may differ from the land race CS used for constructing the draft genome sequence. These variety-specific genes may help genetic analysis of the traits unique to Xiaoyan 81 in the future.

An interesting observation in this work was that subgenome B seemed to host more of the 3026 newly annotated loci than subgenomes A and D (Fig. 2). This may be related to the fact that the chromosomes of subgenome B are generally larger in size than those of A and D subgenomes in common wheat [24, 50].

### Implications for further studies on common wheat grain transcriptome

Several past studies have investigated common wheat transcriptome using HiSeq technology, with substantial insights gained into the number of genes expressed, potential existence of subgenome dominance, and major biological processes operated in the developing grains [33, 51, 52]. Compared to previous studies, our work is unique in using PacBio sequencing to characterize the transcripts in the unfertilized caryopses and developing grains. The newly found chromosomal loci and transcripts, as outlined and discussed above, will contribute positively to further studies on common wheat grain transcriptome. Also valuable is the list of the 6030 genes showing differentially regulated transcriptional pattern during caryopsis development, because many of them have functionally important homologs in model plants (Additional file 9). Moreover, we demonstrated that the differentially regulated isoform expression pattern computed for these genes could be verified using two representatives (Fig. 4). Therefore, the 6030 genes might provide some useful clue for studying the involvement of alternative splicing in regulating common wheat grain development. However, we acknowledge that the identification of this set of differentially expressed genes is preliminary because the transcripts yielded by our PacBio sequencing are limited in number, and the differentially expressed transcripts were judged based on only their presence or absence at the four developmental stages. A detailed identification of the differentially expressed genes based on statistical comparison of their transcript levels at different wheat grain developmental stages is an important target for our future study.

The finding of full-length transcript sequence information for 72 transcribed gluten gene members should be of practical value for more systematically dissecting and improving the function of gluten proteins in common wheat. Of special interest is the identification of full-length transcripts for 52 gliadin gene members, since they are involved in controlling both the processing and nutritional qualities of common wheat, and yet the expression and function of individual gliadin proteins remain poorly understood [53]. With the available full-length transcript information, it is now possible to conduct a detailed proteogenomic analysis to accurately establish the correspondence between gliadin proteins and their coding genes. The resultant data should speed up the identification of functionally important gliadin members, thus aiding the enhancement of common wheat end-use and nutritional qualities through appropriate molecular breeding strategies.

## Conclusions

The data described and discussed above suggest that our PacBio transcript sequencing has generated novel resource and information with positive implications for common wheat genome annotation and gene function research. Clearly, PacBio transcript sequencing can facilitate the annotation and functional studies of complex plant genomes. Our study, together with those published previously [16–20], may help to stimulate more intensive application of PacBio sequencing in plant transcriptome research.

## Methods

### Plant material

Xiaoyan 81 was cultivated in the field as described previously [35]. After heading, the plants were inspected regularly to record the timing of anthesis and grain development. Unfertilized caryopses were collected from 10 different main stem spikes approximately 2 days before anthesis. The grain samples were similarly collected at 5, 15 and 25 DAA, respectively. Total RNA samples were isolated from unfertilized caryopses and developing grains using a commercial Kit (Takara Biotechnology, Dalian, China). The purified RNA was dissolved in RNase-free water, with genomic DNA contamination removed using TURBO DNase I (Promega, Beijing, China). The integrity of the RNA thus prepared was determined with the Agilent 2100 Bioanalyzer (Agilent Technologies, Palo Alto, California). Only the total RNA samples with RIN value ≥ 8 were used for constructing the cDNA libraries in PacBio or HiSeq sequencing.

### PacBio library construction and sequencing

Total RNA (10 μg) was reversely transcribed into cDNA using the SMARTer PCR cDNA Synthesis Kit that has been optimized for preparing high-quality, full-length cDNAs (Takara Biotechnology, Dalian, China), followed by size fractionation using the BluePippin™ Size Selection System (Sage Science, Beverly, MA). Each SMRT bell library was constructed using 500 ng size-selected cDNA with the Pacific Biosciences DNA Template Prep Kit 2.0. The binding of SMRT bell templates to polymerases was conducted using the DNA/Polymerase Binding Kit P5 and v2 primers. Sequencing was carried out on the Pacific Bioscience RS II platform using C3 reagents with 120 min movies.

### Illumina library construction and sequencing

HiSeq libraries were prepared using the Illumina Tru-Seq RNA sample Prep kit. Briefly, fragmentation buffer was added to break mRNA into fragments of 200–700 nucleotides. The resultant mRNA fragments were used as templates to synthesize first strand cDNA. After second strand cDNA synthesis, the fragments with suitable size were gen-purified and amplified by PCR. The PCR products were sequenced using Illumina HiSeq 2000.

### Subread processing and error correction

Effective subreads were obtained using the P_Fetch and P_Filter function (parameters: miniLength = 50, readScore = 0.75, artifact = −1000) in the SMRT Analysis software suite (http://www.pacificbiosciences.com/devnet/). CCS was obtained from the P_CCS module using the parameter MinCompletePasses = 2 and MinPredictedAccuracy = 0. After examining for poly(A) signal and 5′ and 3′ adaptors, only the CCS with all three signals was considered as a FLNC read [20]. Unmerged subreads were also examined for the three signals, and those with three signals were incorporated into the final FLNC read set. Additional nucleotide errors in FLNC reads were corrected using the Illumina RNA-seq data with the software proovread [36].

### Mapping of FLNC reads to CS draft genome sequence

The error corrected FLNC reads were mapped to the draft genome sequence of CS using GMAP as described previously [38]. We used the no-chimera setting to ensure mapping on the same contig as much as possible. The best mapped locus was chosen for each FLNC read based on both identity and coverage values. The genome mapping results of FLNC reads were visualized using the Integrative Genome Viewer [54]. The proportion of FLNC reads carrying complete ORF was evaluated using 5495 wheat full-length cDNAs (downloaded from http://trifldb.psc.riken.jp/v3/index.pl) as reference and by mapping to CS draft genome sequence with the aid of GMAP.

### Verification of splice junctions in G1 FLNC reads

The FLNC reads were aligned to CS draft genome sequence using GMAP, and clustered by locus. The splice junction sites were then obtained with an in-house perl script. In the meantime, the HiSeq transcriptomic reads were also mapped to the draft genome sequence of CS, with the splice junctions identified using Tophat2 [55]. The splice junctions revealed by Tophat2 were compared to those in the FLNC reads to determine if the junction sequence in the concerned FLNC read was supported by HiSeq reads.

### Finding the FLNC read showing cross-contig mapping

The FLNC read exhibiting cross-contig mapping was found using two criteria, 1) positive alignment (with identity and coverage values both higher than 90 %) within 600 bp of CS contig ends, and 2) the orthologs of the concerned contigs were contiguous in the D^t^ (*Ae. tauschii*) or A^u^ (*T. urartu*) reference genomes.

### Discovering the transcript spanning two or more CS chromosomal loci

The transcript that covered two or more loci annotated by CS draft genome sequence was identified using the following standards. First, the transcript showed unique and positive mapping (with identity and coverage values both higher than 90 %) to the exons of the concerned CS loci, and these loci were located on the same contig. Second, the transcript could be reliably mapped (with identity and coverage values both higher than 80 %) to an orthologous genomic region of *B. distachyon* or rice that had only one gene locus annotated.

### Analysis of the genes and their transcripts expressed during caryopsis development

The HiSeq transcriptomic data (Additional file 2) were used to calculate gene expression level and for computing the number of genes expressed at the four caryopsis developmental stages of Xiaoyan 81 (S1 to S4, Table 2). In brief, the four sets of clean reads from the unfertilized caryopses and the developing grain samples at 5, 15 and 25 DAA (Additional file 2) were each mapped to CS draft genome sequence using TopHat2 with default parameters. HTSeq-count was used to determine the reads mapped to individual genes [56]. The gene expression level, as measured by reads per kilobase per million mapped reads (RPKM), was calculated as total exon reads/mapped reads in millions × exon length in kb [56]. A gene was considered as expressed if its RPKM value was > 1. The number of genes with coverage by PacBio sequencing identified transcripts was calculated by comparing the gene sets expressed at S1 to S4 to the 22,768 unique transcripts (Table 1).

The genes covered by PacBio sequencing identified transcripts were divided into two classes, one with a single form of transcript (named as ‘a’) and the other with multiple transcript isoforms (designated alphabetically) (Additional file 9). The expression status of the different types of transcripts at S1 to S4 was checked by finding the HiSeq transcriptomic read(s) that were mapped to transcript-specific splice junction(s), with positive expression judged when the target junction was covered by ≥ 1 HiSeq read. In this way the 6030 genes with differentially expressed transcripts during caryopsis development were compiled (Additional file 9).

For verifying the expression patterns of the two transcript isoforms (a and b) of *TRAES_1DS_114C78BF4* by RT-PCR, three nucleotide primers were designed, FP (5′-ACCACCACCACCTCATTCAA-3′), RP1 (5′-ACATCAAGGGGAGACATGGA −3′) and RP2 (5′-GCAACCCTTCTGTCATCCAC-3′). FP and RP1 were used for amplifying isoform a, whereas FP and RP2 were for isoform b. Total RNA samples from the unfertilized caryopses and developing grains collected at 5, 15 and 25 DAA were reverse-transcribed into cDNA as described above. The resultant cDNA samples were normalized by amplifying a common wheat *actin* gene (GenBank accession AB181991) as detailed previously [57]. Subsequently, the normalized cDNA samples were used for amplifying isoforms a and b, respectively. The PCR was carried out in 20 μl volume containing 10 mM dNTPs, 5 pmol of each primer, and 1 U Taq polymerase (TransGen Biotech, Beijing, China). The cycling parameters included 94 °C for 5 min and 30 cycles of 94 °C for 30 s, 58 °C for 30 s and 72 °C for 1 min, with a final extension at 72 °C for 10 min. The PCR products were separated in 1 % agarose gels. Three independent experiments were conducted with identical results obtained.

### Searching full-length transcripts for gluten gene members

The 197,709 FLNC reads were employed to search for the full-length transcripts of the gluten gene members expressed in Xiaoyan 81 grains by local BlastN. The query sequences used for the search were indicated in Additional file 10. The alignment of three *1Ay* alleles (one from Xiaoyan 81 and two from GenBank, Additional file 11) was carried out using Clustal Omega with default settings in European Bioinformatics Institute website (http://www.ebi.ac.uk/Tools/msa/clustalo/).

### Availability of supporting data

The 197,709 FLNC reads and the HiSeq transcriptomic reads generated in this study have been submitted to the BioProject database of National Center for Biotechnology Information (accession number PRJNA285723).

## Additional files

Additional file 1:
**Summary of the cDNA libraries sequenced by PacBio RSII and the full-length non-chimeric reads obtained.** (DOCX 40 kb) Additional file 2:
**Summary of Illumina HiSeq 2000 transcriptomic reads obtained for unfertilized wheat caryopses and the grains at 5, 15 and 25 days after anthesis (DAA).** (DOCX 39 kb) Additional file 3:
**Analysis of the G2 FLNC reads showing multiple best alignments in the draft genome sequence of common wheat.** (XLSX 799 kb) Additional file 4:
**List of 290 FLNC reads showing cross-contig mapping.** (XLS 40 kb) Additional file 5:
**Mapping data of G4 and G5 FLNC reads in the A** ^**u**^ **genome of** ***T. urartu*** **and D** ^**t**^ **genome of** ***Ae. tauschii*** **.** (DOCX 39 kb) Additional file 6:
**List of 3026 newly discovered loci and their annotations.** (XLS 266 kb) Additional file 7:
**Summary of 22,768 unique transcripts represented by 91,881 high-quality G1 FLNC reads.** (XLS 1122 kb) Additional file 8:
**List of 180 transcripts each spanning two or three separately annotated gene models in Chinese Spring draft genome sequence.** (XLS 28 kb) Additional file 9:
**List of 6030 genes with differentially expressed transcripts during caryopsis development.** (XLSX 546 kb) Additional file 10:
**Finding of full-length gluten gene transcripts and their representative FLNC reads.** (DOCX 26 kb) Additional file 11:
**Comparison of coding region nucleotide sequence of three** ***1Ay*** **alleles.** The *1Ay* sequence of Xiaoyan 81 (represented by 1Ay_XY81) was based on the representative FLNC read identified for *1Ay* in this work (Additional file 10). The sequences of the GenBank accessions AY260548 and AY303766 are two *1Ay* alleles from tetraploid durum wheat and hexaploid spelta wheat, respectively. Asterisks indicate identical nucleotides. The premature stop codons in the coding region of the three sequences are boxed in red. (DOCX 1265 kb)

## Abbreviations

BAC: Bacterial artificial chromosome
bp: Base pair
CCS: Circular consensus sequence
CLR: Continuous long read
CS: Chinese Spring
DAA: Day after anthesis
FLNC: Full-length non-chimeric
GO: Gene ontology
HMW-GS: High-molecular-weight glutenin
kb: Kilobase
KEGG: Kyoto encyclopedia of genes and genomes
KOG: Eukaryotic orthologous group
LMW-GS: Low-molecular-weight glutenin
NR: Non-redundant
ORF: Open reading frame
PacBio: Pacific Biosciences
RIN: RNA integrity number
RPKM: Reads per kilobase per million mapped reads
SGS: Second generation sequencing
SMM: Split-mapped molecule
SMRT: Single-molecule-real time
