# Supplementary material for: Single-molecule real-time transcript sequencing facilitates common wheat genome annotation and grain transcriptome research
Source: BMC Genomics. 2015 Dec 9;16:1039. doi: 10.1186/s12864-015-2257-y (PMC4673716; doi:10.1186/s12864-015-2257-y)
Supplement: Additional file 1: — Summary of the cDNA libraries sequenced by PacBio RSII and the full-length non-chimeric reads obtained. (DOCX 40 kb) [file 12864_2015_2257_MOESM1_ESM.docx]

**Additional file 1:** Summary of the cDNA libraries sequenced by PacBio RSII and the full-length non-chimeric (FLNC) reads obtained

| **Library sequenced** | **Number of continuous long read (CLR)** | **Type I CLR** | | | **Type II CLR** | | **Maximum FLNC read length (kb)** | **Mean FLNC read length (kb)** |
| --- | --- | --- | --- | --- | --- | --- | --- | --- |
|  |  | **Subread** | **Circular consensus sequence** | **FLNC read** | **Subread** | **FLNC read** |  |  |
| < 2 kb | 265,832 | 912,566 | 134,702 | 91,693 | 304,150 | 11,618 | 13 | 1.7 |
| ≥ 2 kb | 261,083 | 705,834 | 105,610 | 83,682 | 266,143 | 10,716 | 25 | 2.8 |
| Total | 526,915 | 1,618,400 | 240,312 | 175,375 | 570,293 | 22,334 |  |  |
