# Supplementary material for: Single-molecule real-time transcript sequencing facilitates common wheat genome annotation and grain transcriptome research
Source: BMC Genomics. 2015 Dec 9;16:1039. doi: 10.1186/s12864-015-2257-y (PMC4673716; doi:10.1186/s12864-015-2257-y)
Supplement: Additional file 2: — Summary of Illumina HiSeq 2000 transcriptomic reads obtained for unfertilized wheat caryopses and the grains at 5, 15 and 25 days after anthesis (DAA). (DOCX 39 kb) [file 12864_2015_2257_MOESM2_ESM.docx]

**Additional File 2:** Summary of Illumina HiSeq 2000 transcriptomic reads obtained for unfertilized wheat caryopses and the grains at 5, 15 and 25 days after anthesis (DAA)

| **Sample** | **Unfertilized caryopses** | **Grains at 5 DAA** | **Grains at 15 DAA** | **Grains at 25 DAA** | **Mean** |
| --- | --- | --- | --- | --- | --- |
| Total number of clean reads | 58,666,676 | 76,489,616 | 62,884,654 | 79,064,836 | 69,276,445 |
| Number of aligned reads | 35,198,773 | 42,093,504 | 29,059,603 | 34,590,257 | 35,235,534 |
| Number of uniquely aligned reads | 28,749,267 | 33,778,419 | 23,637,978 | 29,261,280 | 28,856,736 |

Note: The numbers of aligned reads and uniquely aligned reads were computed after comparing the clean reads to the draft genome sequence of Chinese Spring.
