# Supplementary material for: Single-molecule real-time transcript sequencing facilitates common wheat genome annotation and grain transcriptome research
Source: BMC Genomics. 2015 Dec 9;16:1039. doi: 10.1186/s12864-015-2257-y (PMC4673716; doi:10.1186/s12864-015-2257-y)
Supplement: Additional file 5: — Mapping data of G4 and G5 FLNC reads in the A u genome of T. urartu and D t genome of Ae. tauschii . (DOCX 39 kb) [file 12864_2015_2257_MOESM5_ESM.docx]

**Additional file 5:** Mapping data of G4 and G5 FLNC reads in the A^u^ genome of *T. urartu* and D^t^ genome of *Ae. tauschii*

|  | **FLNC read** | **Mapping results in *T. urartu*** | | **Mapping results in *Ae. tauschii*** | |
| --- | --- | --- | --- | --- | --- |
|  |  | **FLNC reads positively mapped** | **Number of corresponding loci** | **FLNC reads positively mapped** | **Number of corresponding loci** |
| G4 | 8,669 | 3,076 | 748 | 2,150 | 459 |
| G5 | 1,526 | 463 | 224 | 368 | 176 |

Note: Positive mapping was judged based on coverage and identity thresholds both ≥ 90%.
