# Supplementary material for: Single-molecule real-time transcript sequencing facilitates common wheat genome annotation and grain transcriptome research
Source: BMC Genomics. 2015 Dec 9;16:1039. doi: 10.1186/s12864-015-2257-y (PMC4673716; doi:10.1186/s12864-015-2257-y)
Supplement: Additional file 10: — Finding of full-length gluten gene transcripts and their representative FLNC reads. (DOCX 26 kb) [file 12864_2015_2257_MOESM10_ESM.docx]

**Additional file 10:** Finding of full-length gluten gene transcripts and their representative FLNC reads

| **Gluten gene family** | **Full-length transcript found** | **Size of representative FLNC read (bp)** | **Presence of open reading frame** | **Amino acids encoded** |
| --- | --- | --- | --- | --- |
| HMW-GS | *1Ax1* | 2770 | + | 830 |
|  | *1Ay* | 2083 | - | ---- |
|  | *1Bx14* | 2622 | + | 795 |
|  | *1By15* | 2346 | + | 717 |
|  | *1Dx2* | 2795 | + | 839 |
|  | *1Dy12* | 2212 | + | 658 |
| LMW-GS | *Glu-A3-1* | 960 | + | 304 |
|  | *Glu-A3-2* | 1407 | + | 358 |
|  | *Glu-A3-3* | 1093 | + | 376 |
|  | *Glu-A3-4* | 1637 | - | ---- |
|  | *Glu-B3-1* | 1305 | + | 350 |
|  | *Glu-B3-2* | 1428 | + | 392 |
|  | *Glu-B3-3* | 1425 | + | 392 |
|  | *Glu-D3-1* | 1329 | + | 365 |
|  | *Glu-D3-2* | 1298 | + | 354 |
|  | *Glu-D3-3* | 1204 | + | 307 |
|  | *Glu-D3-4* | 1136 | + | 298 |
|  | *Glu-D3-5* | 1674 | - | ---- |
|  | *Glu-D3-6* | 1279 | + | 350 |
|  | *Glu-D3-7* | 1140 | + | 303 |
| Gliadin | *α/β-1* | 1140 | + | 293 |
|  | *α/β-2* | 1144 | + | 293 |
|  | *α/β-3* | 1121 | + | 287 |
|  | *α/β-4* | 1129 | + | 287 |
|  | *α/β-5* | 1131 | + | 286 |
|  | *α/β-6* | 1123 | + | 289 |
|  | *α/β-7* | 1111 | + | 286 |
|  | *α/β-8* | 1098 | + | 296 |
|  | *α/β-9* | 1129 | - | ---- |
|  | *α/β-10* | 1120 | - | ---- |
|  | *α/β-11* | 1643 | - | ---- |
|  | *α/β-12* | 1628 | - | ---- |
|  | *α/β-13* | 989 | - | ---- |
|  | *α/β-14* | 977 | - | ---- |
|  | *α/β-15* | 1124 | - | ---- |
|  | *α/β-16* | 1280 | + | 325 |
|  | *α/β-17* | 1151 | + | 299 |
|  | *α/β-18* | 1143 | + | 296 |
|  | *α/β-19* | 1135 | + | 296 |
|  | *α/β-20* | 1175 | + | 296 |
|  | *α/β-21* | 1151 | + | 296 |
|  | *α/β-22* | 1205 | + | 313 |
|  | *α/β-23* | 1195 | + | 313 |
|  | *α/β-24* | 1038 | + | 297 |
|  | *α/β-25* | 1192 | + | 312 |
|  | *α/β-26* | 1110 | + | 282 |
|  | *α/β-27* | 1235 | + | 286 |
|  | *α/β-28* | 1163 | + | 293 |
|  | *α/β-29* | 1206 | + | 308 |
|  | *α/β-30* | 1184 | + | 309 |
|  | *α/β-31* | 1135 | + | 291 |
|  | *α/β-32* | 1167 | + | 299 |
|  | *γ-1* | 1281 | + | 339 |
|  | *γ-2* | 1103 | + | 285 |
|  | *γ-3* | 1125 | + | 285 |
|  | *γ-4* | 1354 | + | 357 |
|  | *γ-5* | 1169 | + | 302 |
|  | *γ-6* | 1163 | + | 297 |
|  | *γ-7* | 1169 | + | 291 |
|  | *γ-8* | 1186 | + | 326 |
|  | *γ-9* | 1255 | + | 316 |
|  | *γ-10* | 1119 | + | 298 |
|  | *γ-11* | 1145 | + | 295 |
|  | *γ-12* | 1164 | + | 297 |
|  | *γ-13* | 1119 | - | ---- |
|  | *γ-14* | 1472 | - | ---- |
|  | *ω-1* | 1348 | + | 359 |
|  | *ω-2* | 1487 | + | 406 |
|  | *ω-3* | 1555 | + | 439 |
|  | *ω-4* | 1529 | - | ---- |
|  | *ω-5* | 1409 | + | 387 |
|  | *ω-6* | 1271 | - | ---- |

Note: The LMW-GS, α/β-, γ- and ω-gliadin gene members were tentatively named for the purpose of this study. The total number of full-length gluten gene transcripts found by the search was 72.

Information about query sequences: For searching the transcripts of HMW-GSs, the queries were *1Ax1*, *1Ay*, *1Bx14*, *1By15*, *1Dx2* and *1Dy12* sequences (GenBank accessions X61009, JQ689003, AY367771, DQ086215, X03346 and X03041, respectively). For searching the transcripts of LMW-GSs, the query sequences were *LMW-GS* gene members previously identified for the common wheat variety Xiaoyan 54 (GenBank accessions FJ755302 to FJ755316). For searching the transcripts of α/β-, γ- and ω-gliadins, the query sequences were previously identified *α/β-gliadin* (GenBank accessions BQ807130, BQ806209, BQ805841, BQ807194, BQ838853, K03074, DQ40169 and EU680852), *γ-gliadin* (GenBank accessions AAD30556, JX295577, FJ006629 and AF234643) or *ω-gliadin* (GenBank accessions BQ804665, BQ838934, BQ804424, BQ805896, BQ806240, AAT74547 and ACN96903) gene members.
