# Supplementary material for: Single-molecule real-time transcript sequencing facilitates common wheat genome annotation and grain transcriptome research
Source: BMC Genomics. 2015 Dec 9;16:1039. doi: 10.1186/s12864-015-2257-y (PMC4673716; doi:10.1186/s12864-015-2257-y)
Supplement: Additional file 11: — Comparison of coding region nucleotide sequence of three 1Ay alleles. The 1Ay sequence of Xiaoyan 81 (represented by 1Ay_XY81) was based on the representative FLNC read identified for 1Ay in this work (Additional file 10). The sequences of the GenBank accessions AY260548 and AY303766 are two 1Ay alleles from tetraploid durum wheat and hexaploid spelta wheat, respectively. Asterisks indicate identical nucleotides. The premature stop codons in the coding region of the three sequences are boxed in red. (DOCX 1265 kb) [file 12864_2015_2257_MOESM11_ESM.docx]

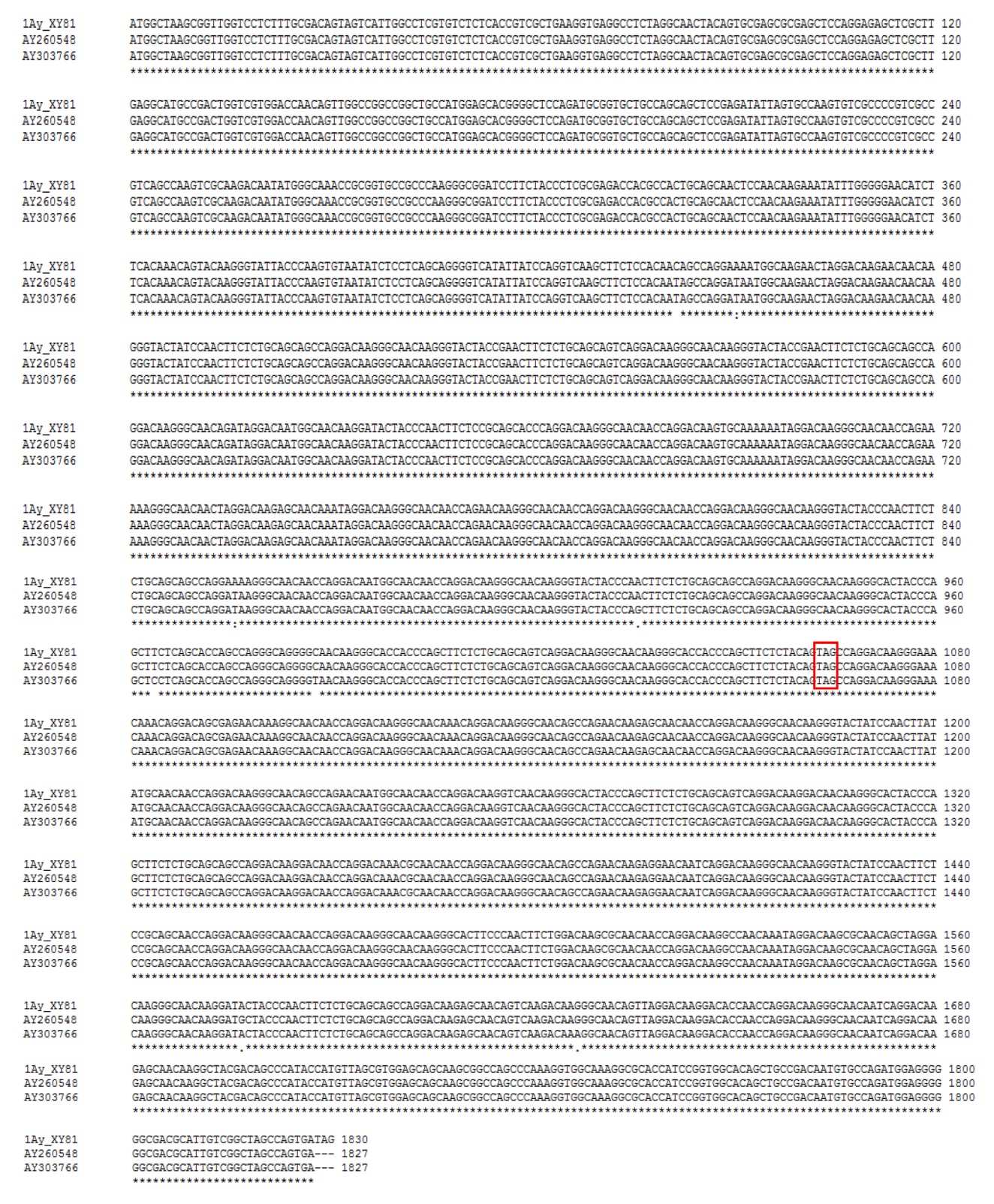


**Additional file 11:** Comparison of coding region nucleotide sequence of three *1Ay* alleles. The *1Ay* sequence of Xiaoyan 81 (represented by 1Ay_XY81) was based on the representative FLNC read identified for *1Ay* in this work (Additional file 10). The sequences of the GenBank accessions AY260548 and AY303766 are two *1Ay* alleles from tetraploid durum wheat and hexaploid spelta wheat, respectively. Asterisks indicate identical nucleotides. The premature stop codons in the coding region of the three sequences are boxed in red.
